# Synchronous presentation of muscle-invasive urothelial carcinoma of bladder and peritoneal malign mesothelioma

**DOI:** 10.1590/S1677-5538.IBJU.2018.0815

**Published:** 2019-09-02

**Authors:** Cem Basatac, Fatma Aktepe, Sezer Sağlam, Haluk Akpınar

**Affiliations:** 1 Department of Urology, Istanbul Bilim University, Istanbul, Turkey;; 2 Department of Pathology, Group Florence Nightingale Hospitals, Istanbul, Turkey;; 3 Department of Medical Oncology, Istanbul Bilim University, Istanbul, Turkey

**Keywords:** Mesothelioma, Peritoneum, Neoplasms

## Abstract

**Introduction:**

Cancer is one of the most important leading cause of death in man and woman in the world. The occurrence of new cancer has become more frequent in recent years due to strict screening protocols and occupational and environmental exposure to carcinogens. The incidence of secondary malignancies has also increased due to close medical follow-up and advanced age. Herein, we report a case and its management diagnosed as synchronous peritoneal malignant mesothelioma and muscle-invasive urothelial carcinoma.

**Case Description:**

A 71-year-old male presented with macroscopic hematuria and abdominal distension increasing gradually. A contrast enhanced computerized tomography demonstrated bladder mass and diffuse ascites with nodular peritoneal thickening and umbilical mass. He was treated with the multidisciplinary team working including urologist, medical oncologist and general surgeon.

**Conclusions:**

To our knowledge, this is the first case of peritoneal malign mesothelioma with synchronous muscle-invasive urothelial carcinoma. Because of the rarity of this condition, there is still no consensus on the definitive treatment protocols, yet. Individualized treatment with multidisciplinary close follow-up might improve the survival outcomes.

## INTRODUCTION

Malign mesothelioma is a relatively rare cancer that arises from mesothelial cells of pleura, peritoneum, pericardium, and tunica vaginalis. Primary peritoneal mesothelioma (PM) accounts for 10% to 30% of all cases of malignant mesothelioma (1). Even though asbestos exposure plays an important role in the development of tumor cells, PM is rarely associated with asbestos exposure and tends to present at the younger age (2, 3). On the other hand, bladder cancer is one of the most common urologic malignancies. It was the 4^th^ and 12^th^ most frequent malignant tumor among men and women, respectively (4). Transitional cell cancer of bladder is the most common form and it accounts for about 90% of cases (5). Advanced age and smoking are identified as the most important independent risk factors for the development of this tumor. Although the incidence of the secondary malignancy in patients who have bladder cancer increase with the advanced age and longer follow-up, invasive urothelial carcinoma and synchronous malignant PM is a very rare entity at the current literature. In this case, we aim to report this unusual case of muscle-invasive bladder cancer with synchronous peritoneal malign mesothelioma and discuss its management and treatment alternatives.

## CASE DESCRIPTION

**Figure 1:**
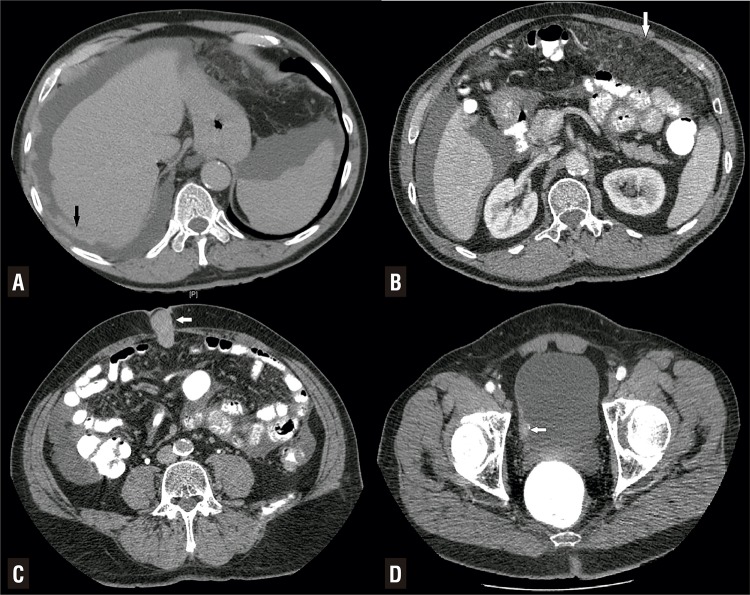
A) Peritoneal thickening on the right hemidiaphragm (black arrow). B) Diffuse omental invasion of peritoneal mesothelioma (omental caking, white arrow). C) CT imaging of umbilical mass (white arrow) D) Bladder mass with sessile growth pattern (white arrow).

**Figure 2:**
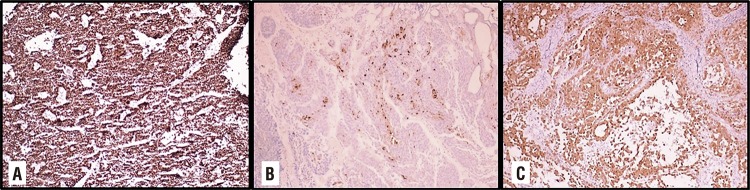
A) Immunohistochemical GATA positivity in urothelial carcinoma cells. B) Immunohistochemical focal uroplakin III positivity in urothelial carcinoma cells. C) Immunohistochemically, diffuse positivity with calretinin in mesothelioma cells.

A seventy-one-year-old male patient without any history of asbestos exposure has admitted to our hospital with complaints of macroscopic hematuria and abdominal distension increasing gradually. In his medical history, he underwent transurethral resection of the prostate due to benign enlargement 5 years ago. Physical examination revealed abdominal distention with flank and shifting dullness. An umbilical nodule was also noted. The urinalysis revealed pyuria and microscopic hematuria, but the urine culture was sterile. An abdominal ultrasonography showed bladder mass measured as 15mm and diffuse intra-abdominal free fluid collection. A computerized tomography (CT) demonstrated diffuse ascites with nodular peritoneal thickening and multiple lymph nodes over 10mm at the internal iliac, common iliac and paraaortic regions. An umbilical mass measured as 30mm was also seen on CT imaging (Figure-1). Surgical treatment in the same session was desired by the patient. After written informed consent was obtained, a transurethral bladder tumor resection and laparoscopic peritonectomy were carried out consisting of the omentum, left and right hemidiaphragm, pelvic peritoneum and umbilical mass. Pathologic examination revealed high-grade urothelial carcinoma with the muscularis propria invasion on the TUR specimen and malign PM on the peritoneal specimens. Immunohistochemistry was used to confirm pathologic diagnoses for both cancers. Immunohistochemically, urothelial carcinoma was widely positively stained with GATA and a focal positive with uroplakin III but no mesothelioma cells were stained. Additionally, mesothelioma cells were strongly and diffusely stained with calretinin, however, urothelial carcinoma cells were not stained (Figure-2). Sixth cycles of pemetrexed, cisplatin, bevacizumab every three weeks were chosen for systemic chemotherapy for the treatment of both cancers. Patient tolerated well systemic chemotherapy and a CT scan showed no evidence of disease progression at the sixth months follow-up visit. After the interview with the patient about treatment options, hyperthermic intraperitoneal chemotherapy (HIPEC) with cisplatin and doxorubicine was planned for peritoneal malign mesothelioma. He is still monitored closely by the multidisciplinary team consisting of the urologist, medical oncologist, and general surgeon.

## DISCUSSION

Peritoneal mesothelioma is a rare variant of malignant mesotheliomas. The prognosis depends primarily on the type of tumor. Localized and well-differentiated PM tends to be clinically indolent with survival in the range of years and even decades. However, diffuse and high-grade tumors are more destructive and infiltrative with a median survival time of less than 1 year (6). Several histologic parameters have been identified as predictors of worse prognosis. These are depth of invasion, mitotic count, nuclear grade, sarcomatoid differentiation, desmoplastic and lymphoid response, and lymph node metastasis (7). Although systemic chemotherapy has historically been the treatment of choice, there is still no certain consensus about optimal treatment of PM due to lack of comparative and high-quality studies.

Treatment alternatives for PM include observation, chemotherapy alone, cytoreductive surgery (CRS) alone, CRS combined with chemotherapy and CRS combined with HIPEC. One of the largest studies by Verma et al. suggested that combined modalities such as CRS/chemo and CRS/HIPEC had the most favorable results in terms of overall survival rates (8). On the contrary, treatment of muscle-invasive bladder cancer is well known at present. Definitive treatments are radical cystectomy with suitable urinary diversion and external beam radiation therapy for the organ-confined disease. Bladder-sparing modalities with trimodality therapy consisting of deep transurethral resection of bladder tumor combined with chemo and radiotherapy can be also considered in selected cases. Systemic chemotherapy with platinum-based regimes remains the first-line treatment for the advanced bladder cancer (9). In the presented case, we were not willing to perform radical cystectomy due to synchronous diagnose of PM.

Synchronous primary cancer with peritoneal malign mesothelioma is an extremely rare condition. There are only a few reports related to this topic in the current literature. Jacobsen et al. reported a case of PM in a patient with non-muscle invasive urothelial carcinoma (10). The other published case reports only consist of gynecologic cancers (11). One of the largest studies by Attenoos et al. reported that incidence of secondary malignancy after malign mesothelioma as 1-2% at post-mortem examination. However, none of these were urogenital tumors (12). Additionally, Chen et al. reported that the incidence of renal cell carcinoma has increased in patients with malign mesothelioma. But they didn’t declare any significant etiologic and genetic association among these tumors (13). On the other hand, the most frequent secondary malignancies after bladder cancer were as follows: lung, prostate and breast cancer (14). Increased risk of secondary tumors may be attributable due to smoking and close medical follow-up after primary diagnose of the bladder tumor.

In conclusion, to our knowledge, this is the first case published in the literature who have muscle-invasive bladder cancer with synchronous peritoneal malign mesothelioma. As in the presented case, precise pathologic examination with correct immunohistochemical staining is essential to confirm the diagnosis and exclude other types of malignancies. In addition, accurate clinical staging of these tumors is needed in order to plan treatment options. However, no curative or definitive treatment is determined at the current literature since its extremely rare condition. Such challenging cases must be addressed through direct communication in a multidisciplinary tumor board setting. Individualized treatment with multidisciplinary close follow-up schemes might improve the survival outcomes in these patients.
